# Therapeutic expectations: Dentistry relies less on dental plaque as a major etiological factor OR On the dental needs of young orthodontic patients (12-20 years old)

**DOI:** 10.1590/2177-6709.21.1.018-023.oin

**Published:** 2016

**Authors:** Alberto Consolaro, Daniele Nóbrega Nardoni, Leopoldino Capelozza, Paulo Henrique X. Franco, José Antônio Z. Cappellozza

**Affiliations:** 1 Full professor, Universidade de São Paulo (USP), Undergraduate program, Bauru, São Paulo, Brazil and Universidade de São Paulo (USP), Graduate program, Ribeirão Preto, São Paulo, Brazil; 2 MSc in Oral Biology, Universidade Sagrado Coração (USC), Bauru, São Paulo, Brazil; 3 Professor of Orthodontics, Universidade Sagrado Coração (USC), Bauru, São Paulo, Brazil; 4 Specialist in Orthodontics, Universidade Sagrado Coração (USC), Bauru, São Paulo, Brazil

**Keywords:** Orthodontic planning, Caries, Periodontal disease, Etiology, Dental trauma

## Abstract

In Brazilian cities and states governed efficiently with wealth ethically administered, carious and periodontal diseases have prevalence rates similar to those found in socially developed European countries. This shift in reality, noticed over the last 15 years, reflects on changes in the etiological factors related to patients' major expectations and needs - especially young and orthodontic patients - which turn out to be a result of dental trauma, malocclusion, facial aspect, dental agenesis and iatrogenesis. Under such conditions, patients begin to appreciate the value of tooth position, color and shape, their smile and function: details become relevant. Carious and periodontal diseases remain an issue, not only from a preventive prospect, but also from a curative one. Nevertheless, it should be noted that changes and development are inevitable, and we should be prepared to contribute to the wellbeing of people, particularly regarding their novel needs and expectations.

## Abstract (pt)

A cárie e a doença periodontal em cidades e estados brasileiros com gestão eficiente e ética dos recursos apresenta índices de prevalência semelhantes aos de países europeus mais desenvolvidos socialmente. Essa mudança de realidade, percebida ao longos dos últimos 15 anos, reflete na mudança dos agentes etiológicos relacionados às principais expectativas e necessidades dos pacientes - especialmente os jovens e ortodônticos -, as quais passam a ser decorrentes do traumatismo dentário, má oclusão, aspecto facial, agenesias dentárias, iatrogenias. Nessa nova condição, passam a valorizar a posição, cor e forma dos dentes, seu sorriso e suas funções: os detalhes passam a ser relevantes. Ainda devemos nos preocupar com a cárie e doença periodontal, inclusive nos seus aspectos curativos e não mais apenas preventivos, mas devemos perceber que as mudanças e evoluções são inevitáveis, e nos preparar para contribuir para o bem das pessoas quanto às suas novas necessidades e expectativas.

Truly developed communities have reduced inequality, full access to health services and laid-back inhabitants, even when they are older. This is because social support will treat them with dignity until their last breath. A number of countries have reached such a stage in which the State exists to take care of people. This is the case of Norway, Sweden, Denmark, Finland, New Zealand, Germany, Belgium, the Netherlands, among others including Uruguay. Brazil made significant progress after the civil and military dictatorship in 1964.

In the 1900s, the average life expectancy of people was 31 years old. It is worth noting that this reality does not go back way to our ancestors, but was the reality of our grandparents and great-grandparents! The most common causes of death were:

1) violence in wars, disputes and slavery;
2) infectious diseases, since vaccines and antibiotics were nonexistent;
3) nutritional insufficiency; and
4) childbirth in poor conditions or unassisted, particularly during pregnancy.

Inflammation is the defense mechanism acting against all types of offending agents: from physical wounds to chemical agents and, most important, microorganisms. Inflammation brings along signs and symptoms: heat and redness, tumor and pain! 

In the last century, vaccines, antibiotics, analgesic and anti-inflammatory drugs were invented to control the process of inflammation, without decreasing inflammation efficiency. In developed communities, inflammation and repair are no longer the primary mechanism that recovers microorganism-damaged tissues, which caused infected people to become incapable or dead. 

Inflammation and repair are now monitored and controlled in lesions and amputations that result from:

1) trauma associated with the transportation of people and goods;
2) occupational, leisure and sports accidents;
3) body reconstruction by cosmetic, orthognathic and other types of surgery for esthetic-functional purposes;
4) surgery for removal of malignant neoplasms, such as breast, prostate, mouth and lung cancer, among others.

Inflammation involves repair which is responsible for reconstructing vascularized connective tissues forming most parts of our body mass, namely: bones, cartilage, muscles, fat and fibrous structures. Inflammation and repair allow and accept placement of prosthetic material as well as material aimed at volume filling and replacement of lost parts. 

Presently, inflammation, as a defense and reconstruction process, will carry immune system cells, or leukocytes, and substances - such as antibodies and mediators usually present in the blood - to damaged body parts in order to promote repair after an attack. In the past, they were incapable of finding microorganisms present at the site due to poor hygiene. Now they find tissue debris, blood clot, areas that must be recovered, as well as occasional, common, easily controlled bacteria. 

Whenever people live more and with higher quality, every detail and function of the body becomes relevant and noticeable! Health care professionals will have to change their paradigms: the offending agents attacking the human body are different now! Retreatment and procedures from ancient times characterized by poor education and hygiene are still employed; however, a new era is envisaged.

Let us look at the past and the future simultaneously! At present, those who study inflammation and repair in the human health field must recall Janus' story.

## The lesson left by Janus!

**Figure 1:**
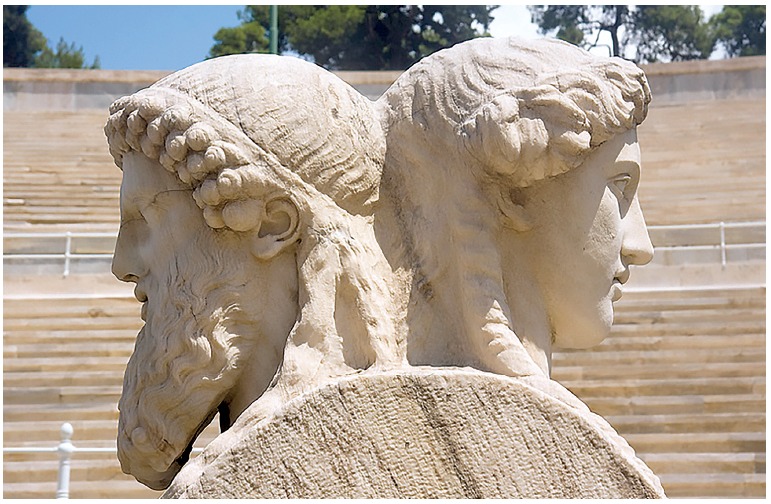
- Janus' statue and his two faces: past and future.

Janus was a Roman god with two faces: one faced towards the past, and the other faced towards the future (Fig 1). His figure is constantly associated with periods of transition. He is considered the god of beginnings of processes, phases and times, the god of decisions and choices that open up new ways: he symbolizes a new era. The month of January is named after Janus!

A statue representing Janus is found at the Vatican, in a Roman monument, since it is believed that there is an association between Janus and the foundation of the city. In ancient mythology, Janus ruled Latium, the region of central Italy, and was responsible for bringing progress to the area after bringing gold, money and agriculture; thus, establishing the beginning of a new era. Janus is said to be the inventor of the wreath, boat, ship and money: many places in Greece and Italy have coins stamped with a two-faced figure.

Janus was the first god worshiped in Roman ceremonies before they were taken over by the Greek. According to myth, Janus was a mortal man who was born in Greece. After he moved to Latium, he engaged in marriage with the queen. After his wife's death, Janus began to rule the area by himself, focusing his administration on transformation, scientific progress, the creation of laws, refinement of cultures and manufacturing of the first coins. All changes that were introduced brought about peace and prosperity to Latium, as it had never experienced before. 

After Janus' death, he was considered the god of transitions due to having a life dedicated to transformation: he looked not only to the past, but also to the future. For this reason, the first month of the year is known as January: in honor to Janus!

## Janus' Dentistry: a stage of transition

From the '90s onward, Brazilian Dentistry has undergone many changes and transformations. Two determining facts might be highlighted: social changes and osseointegrated implants. 

Every day, carious and periodontal diseases increasingly disappear. Every day, Dentistry ceases to deal with problems arising from the action of bacteria, resulting from poor oral hygiene and education. Once people have healthy teeth, they begin to appreciate the value of tooth position, color, and shape, as well as their smile and function: details become relevant!

Dental professionals will have to change their paradigms. Dental issues and therapeutic planning must take into consideration the consequences of trauma, surgical procedures, iatrogenesis and congenital tooth loss. We are in a transitional period; thus, retreatment and procedures from ancient times are still employed; however, a new era is envisaged, bringing along a Dentistry aimed at optimizing existing teeth, instead of extracting them.

Every day, periapical and periodontal pathologies will have surgical procedures, dental trauma and iatrogenesis as their causes: all of which will rarely have the remarkable or decisive presence of bacteria. Meanwhile, it is key to assess, cogitate and correct past changes, based on lack of hygiene and proper education. We are living a transitional period, the era of Janus' Dentistry. 

## Pus as a sign of a large amount of bacteria

Our body has an average of 10 trillion cells comprising communities that form tissues and organs which, in turn, concomitantly perform the functions that keep us alive. Meanwhile, and in contrast, our body has 100 trillion bacteria - almost all of our inner and outer surfaces, comprising the microbiotas. Nevertheless, if we were able to eliminate all bacteria, surprisingly, we would not be able to survive! We need them to live!

Our organism cannot allow direct, open communication with the outer environment. Tissues do not have bacteria moving around freely from the inner to the outer environment and *vice versa*; this will occur in an occasional and brief basis only, as they will survive just for a few seconds within tissues, especially blood. 

Should microbiotas insist in entering bacteria-free tissues, defense mechanisms will be triggered: blood antibodies and proteins are ready to identify them as foreign bodies, thus destroying them immediately - or in an indirect manner by attracting the cells of our immune system towards them, especially within the blood where those cells are also known as leukocytes, white blood cells, inflammatory cells or immune cells.^1^

Human microbiotas are predominantly composed of *staphylococcus* (when bacteria appear round and form in grape-like clusters) and*streptococcus* (when bacteria appear round and form in pearl chain-like clusters). In the blood, leukocytes are predominantly found as neutrophils whose nuclei present with different shapes and, for this reason, are also known as polymorphonuclear leukocytes: they account for 55 to 65% of circulating leukocytes. Bacteria that somehow enter the tissues are usually*staphylococcus* and *streptococcus*, both with a high ability of interacting with neutrophils. Neutrophils basically exist to fight against bacteria entering and acting within tissues, via a highly effective process of phagocytosis.^1^

Interaction between neutrophils and *staphylococcus* as well as*streptococcus* generally results in the formation of pseudopodia surrounding the bacteria, creating a vacuole - or phagosome - where a number of bactericidal and toxic by-products are released: protein-degrading enzymes, or proteolytic enzymes; chlorine solutions and oxygen-derived by-products, such as hydrogen peroxide, all of which are usually stored within neutrophils cytoplasmic vacuoles, coated by resistant micromembranes. However, before phagosomes close up entirely, enclosing the bacteria, neutrophils tend to release their vacuolar content, overflowing or throwing out a considerable portion of toxic by-products into tissues where they had served as immune cells.

Regurgitation of neutrophils phagocytizing *staphylococcus* and*streptococcus* bacteria results in degradation of the connective tissue where they are acting. Exudate or inflammatory edema, which initially resembles a citrine liquid, is now a viscous and yellow fluid, or purulent exudate, full of mediators, bacteria and bacteria by-products. The situation becomes worse when neutrophils, now weak and defeated by bacteria, burst and release their lysosomal or vacuolar content into the area where they are acting.

The formation of pus only exists as a result of neutrophils interacting with*staphylococcus* and *streptococcus*. Thus, any situation indicating the presence of pus suggests the primary presence of bacterial contamination or secondary bacterial contamination. Neutrophils are also known as pyocytes, or pus cells, whereas *staphylococcus* and*streptococcus* bacteria are known as pyogenic (marked by pus production).

## Dentistry free of caries-forming bacteria as the cause of pulp pathologies

In cases with a high prevalence of dental caries, inflammatory processes of the pulp, or pulpitis, are very likely to lead to pus formation, given that the microbiota usually involved in carious processes is predominantly composed of*staphylococcus* and *streptococcus* bacteria. Should caries-associated pulpitis evolve with time, it ends up forming a purulent exudate that not only increases in volume, but also in painful symptoms, promoting pulp necrosis, whether septic or contaminated. Bacteria gradually reach the pulp, via dentin tubules, enter its structure and interact with neutrophils at the site.

Should conventional interventions, whether accidental or not, over the pulp tissue, be performed in an aseptic environment, reduces the risk of pus formation. Asepsis is a set of procedures aimed at preventing microorganisms from entering sites where they did not exit previously. Inflammation triggered by the mechanical action of tools or chemical action of products placed near the underlying pulp might result in little symptoms or no symptoms at all.

Should that be the case, in the absence of bacteria and by-products, and if the products applied are no longer toxic or become little toxic to tissues, discreet and limited inflammation evolves to repair: fibrous connective tissue-based or under odontoblastic resetting, followed by the deposition of repair dentin. 

Should material toxicity remain at site, the inflammatory process might evolve to a transitional stage, with mild to moderate symptoms that are likely to cease if the process gradually evolves to aseptic pulp necrosis. However, it will never evolve to pus formation. The formation of pus suggests bacterial contamination or presence at site.

Some types of material, such as resins, are able to induce chronic inflammation which tend to persist over time, nearly asymptomatic. Other types of material might denaturate pulp tissue proteins and gradually coagulate the entire conjunctiva structure; slowly and nearly asymptomatic as well.

## Dental trauma: the primary cause of tooth loss in developed countries

In Brazilian cities and states where health programs run efficiently and with wealth ethically administered, carious and periodontal diseases have prevalence rates similar to those found in socially developed European countries, considered as good oral health examples. The study recently conducted by Nardoni et al^2 ^reveals this exact reality.

In those places, where oral health resources are applied efficiently and correctly, pulp, periapical and periodontal issues are rarely diagnosed as a result of dental plaque. Pulpitis and pulp necrosis cases result from dental trauma - from minor concussions to tooth fracture and avulsion. They might as well also result from iatrogenisis acting over the dentin-pulp complex. This line of thought extrapolates to periapical and periodontal lesions. 

In modern Dentistry, we should pay closer attention to enamel cracks, enamel splinters and incisal microfractures, in addition to tooth darkening, as resulting from dental trauma. The time during which orthodontic treatment and/or occlusal trauma were responsible for pulp necrosis lays back in the history of our evolution.

At the aforementioned Brazilian cities and states, an endodontist will rarely treat clinical cases of pulp necrosis primary caused by caries. Similarly, the demand for exclusively periodontal services has drastically dropped. Data presented by Nardoni et al^2 ^reinforce the low occurrence of carious teeth (0.09 of caries per tooth). The prevalence of restorations was higher, but still low. Those data reveal very good oral health conditions for the parameters assessed, with no patient presenting signs of endodontic treatment. As regards tooth extraction, 11 teeth were found to be missing, with only one extraction being recommended due to caries. All other ten cases were for orthodontic purposes. The association between little occurrence of caries and its consequences was confirmed by the little occurrence of extractions. Only ten teeth had extraction justified by orthodontic treatment planning. A total of 236 third molars were recommended for extraction.

As for the presence of osseointegrated implants, no implant was found to be present in the sample assessed. This was already expected, based on the age of patients evaluated. The need for having implants placed, in order to replace missing teeth due to agenesis, was present in ten cases after or during orthodontic treatment. A relatively high frequency of dental anomalies was found to affect 42 out of 100 patients. Two or more dental anomalies were identified in 20% of the sample.

## Synthesis and final considerations

The Brazilian are facing a new reality in terms of oral health, and Orthodontics needs to adapt to it. The most prevalent causes of dental and oral problems are dental trauma and malocclusion, while the major reason for patients seeking a dental and/or oral health professional are the color and shape of teeth, in addition to agenesis. 

In this context, not only dental trauma is seen as relevant, but also discrepancies between the maxilla and the mandible, which affect patient's face and occlusion. This also applies to developmental disorders, such as cleft lip and palate and dental agenesis.

Janus' example suggests that we do not forget the past, highlighting those who still face the reality of that time, given that not all Brazilian cities and states have had the opportunity to experience such development due to governmental issues. Carious and periodontal diseases remain an issue, not only from a preventive prospect, but also from a curative one. Nevertheless, it should be noted that changes and development are inevitable, and we should be prepared to contribute to the wellbeing of people, particularly regarding their novel needs and expectations. Denying change and evolution is not wise, since it is inevitable that the new will always come!
